# Determinants of job performance among secondary school teachers in Vietnam: evidence from a mixed-methods study

**DOI:** 10.3389/fsoc.2026.1836374

**Published:** 2026-05-22

**Authors:** Quoc Giang Tran, Thi Tuyet Hanh Nguyen, Tan Dat Truong

**Affiliations:** Dong Thap University, Cao Lanh, Vietnam

**Keywords:** job performance, mixed methods, resilience, school governance, self-efficacy, teacher burnout

## Abstract

**Introduction:**

This study examined how individual and organizational factors are associated with the job performance of secondary school teachers in Vietnam, with particular attention to self-efficacy, resilience, principal support, and burnout.

**Methods:**

An explanatory sequential mixed-methods design was employed. In the quantitative phase, survey data from 536 secondary school teachers were analyzed using descriptive statistics, Pearson correlation, and multiple linear regression. In the qualitative phase, 16 semi-structured interviews were conducted to explain and elaborate the statistical patterns identified in the survey results.

**Results:**

The quantitative findings showed that self-efficacy was the strongest positive predictor of job performance, followed by resilience and principal support, whereas burnout was negatively associated with performance. The qualitative findings provided deeper insight into these relationships. Teachers linked strong performance to pedagogical competence, adaptability, and confidence in managing classroom demands. They also described supportive leadership, peer collaboration, and collegial sharing as important conditions that helped sustain professional functioning. By contrast, burnout was associated mainly with administrative workload and documentation pressures rather than with teaching itself.

**Discussion:**

Taken together, the findings suggest that improving teacher performance in Vietnam requires a dual strategy: strengthening teachers’ professional capacity and restructuring school governance processes to reduce administrative overload and foster more supportive school environments.

## 1 Introduction

In the context of globalization and the ongoing transformation of educational systems, teachers’ job performance has become a central concern for both researchers and policymakers because it reflects, and helps shape, the overall quality and sustainability of schooling. A substantial body of empirical evidence has shown that teachers are a decisive factor influencing students’ academic achievement and the long-term development of schools (Ronfeldt et al., 2013; Seidel and Shavelson, 2007). Across Southeast Asia, education systems are increasingly emphasizing the importance of supportive working environments in which teachers’ psychological well-being and professional functioning are treated as core components of human resource management (Oblina et al., 2021). At the same time, however, teachers are facing growing demands related to accountability, pedagogical innovation, and administrative workload. In Vietnam, these pressures have become particularly visible during the implementation of the 2018 General Education Curriculum. This reform context, combined with the lingering effects of crises such as the COVID-19 pandemic, has exposed weaknesses in teacher support systems and intensified concerns about stress, motivation, and professional performance (Pham et al., 2023).

Teachers’ job performance is not produced by individual effort alone. Rather, it emerges from the interaction between internal psychological resources and the organizational conditions in which teachers work. From the perspective of occupational psychology, self-efficacy and resilience are key resources that help teachers sustain professional engagement under pressure. Previous studies have shown that teachers with stronger beliefs in their instructional and classroom-management capabilities are more likely to maintain higher levels of effectiveness and more stable professional functioning (Burić and Kim, 2021; Canrinus et al., 2012; Klassen and Chiu, 2010; Klassen et al., 2010; Peng and Mao, 2015). Recent validation studies in Vietnam have also provided contextually relevant measurement frameworks for self-efficacy and resilience, opening new opportunities for examining teacher capacity in local settings (Ho et al., 2023; Trang and Thang, 2023).

At the same time, these individual resources do not operate in isolation. From a school governance perspective, principal support and leadership practices shape the organizational climate in which teachers either flourish or struggle. Research on supportive, transformational, and distributed leadership has shown that school leaders can strengthen professional trust, autonomy, and work motivation when they provide clear guidance, open communication, and meaningful delegation (Boyd et al., 2011; Chong et al., 2015; García Torres, 2019; Leithwood and Sun, 2012; Liu and Werblow, 2019; Sun and Xia, 2018; Yao et al., 2020). By contrast, weak managerial support and excessive non-teaching demands contribute to teacher burnout, which undermines performance and increases withdrawal from the profession (Anastasiou and Belios, 2020; Capone and Petrillo, 2020; Molero Jurado et al., 2019; Skaalvik and Skaalvik, 2017).

Despite the richness of this literature, several important gaps remain. First, studies in Vietnam and in comparable educational settings have often examined either enabling factors such as self-efficacy and support, or constraining factors such as burnout, without integrating these elements into a single model of teacher job performance. Second, much of the existing research relies on single-method quantitative designs. Although such studies are useful for identifying general patterns, they often do not explain how institutional culture, leadership practices, and administrative demands shape teachers’ lived experiences and professional functioning. This limitation is particularly important in contexts such as Vietnam, where educational reform is unfolding within a system characterized by high policy intensity, strong administrative accountability, and uneven local conditions. In such contexts, statistical associations alone may not fully explain how teachers interpret professional pressure, how resilience is sustained, or why some organizational environments are more supportive than others.

Importantly, the Vietnamese context is not only locally relevant; it also offers broader analytical value for international scholarship. Vietnam represents a case of a rapidly reforming education system in which teachers are expected to respond simultaneously to curriculum change, performance accountability, and organizational restructuring. Examining teacher job performance in this context can therefore contribute to wider discussions in the international literature on how psychological resources interact with governance conditions in periods of systemic change. More specifically, this study helps clarify how supportive leadership, resilience, and self-efficacy may buffer professional strain, and how administrative burden may weaken performance, in ways that may also be relevant to other centralized, reform-oriented, or resource-constrained educational systems.

To address these gaps, this study adopts an explanatory sequential mixed-methods design (QUAN → qual), which is well suited to research questions that require both pattern identification and contextual interpretation (Creswell and Tashakkori, 2007; Hanson et al., 2005; Ivankova et al., 2006; Johnson and Onwuegbuzie, 2004; Johnson et al., 2007). The quantitative phase is used to examine the predictive relationships between key psychological and organizational factors and teachers’ job performance, while the qualitative phase is employed to provide deeper insight into how teachers perceive and experience these relationships in practice. In this way, the mixed-methods approach serves not as an end in itself, but as a means of generating a more comprehensive understanding of teacher performance within a reforming educational context.

Accordingly, the primary objective of this article is to identify, measure, and interpret how self-efficacy, resilience, principal support, and burnout are associated with the job performance of secondary school teachers in Vietnam. By doing so, the study seeks to contribute to the international literature on educational work, occupational psychology, and school governance, while also offering practical insights for policymakers and school leaders seeking to strengthen teacher support systems and improve the sustainability of school reform.

To guide the integrated data collection and analysis process, this study addresses the following research questions:

*RQ1*: To what extent do self-efficacy, principal support, resilience, and teacher burnout predict the job performance of secondary school teachers in Vietnam?

*RQ2*: How do teachers perceive and experience the role of school governance culture and organizational support networks in sustaining teaching motivation and responding to professional pressures?

*RQ3*: How do teachers’ narratives and lived experiences help explain the statistical relationships identified in the quantitative model?

## 2 Literature review and conceptual framework

### 2.1 Educational reform and teacher work in the Vietnamese context

Vietnam’s secondary education system is currently undergoing substantial transformation under the 2018 General Education Curriculum reform. This reform requires teachers not only to update instructional content and pedagogical approaches, but also to adapt to new expectations regarding competency-based teaching, student assessment, professional accountability, and school-level implementation processes. In practice, these changes have expanded the scope of teachers’ work beyond classroom instruction, as they must simultaneously respond to curriculum innovation, reporting requirements, professional development demands, and increasing expectations from school leadership and education authorities. In such a context, teacher job performance cannot be understood solely as an individual attribute; it is shaped by the interaction between teachers’ internal professional resources and the organizational conditions under which reform is implemented.

This Vietnamese context is also analytically significant beyond the national setting. As in many centralized and reform-oriented education systems, teachers in Vietnam are positioned at the intersection of policy change, institutional expectations, and everyday school realities. Examining teacher performance under these conditions can therefore contribute to broader discussions about how psychological resources such as self-efficacy and resilience interact with governance-related conditions such as principal support and administrative pressure. In this sense, Vietnam provides a useful case for understanding how teachers sustain performance during periods of systemic change, particularly in contexts where reform intensity is high and organizational demands may outpace available support structures.

### 2.2 Self-efficacy and job performance

Self-efficacy refers to teachers’ beliefs in their capacity to organize and execute the actions required to accomplish teaching tasks effectively. In educational settings, this construct has consistently been associated with classroom management, instructional quality, persistence in the face of difficulty, and professional confidence (Klassen and Chiu, 2010). International evidence suggests that self-efficacy is closely related to broader indicators of professional identity, motivation, and work functioning (Burić and Kim, 2021; Canrinus et al., 2012; Klassen et al., 2010; Peng and Mao, 2015; Stephanou et al., 2013). In addition, teachers’ sense of efficacy has been associated with more positive perceptions of work and stronger professional engagement across contexts (Buonomo et al., 2020; Liu et al., 2018).

Within Vietnam, the relevance of self-efficacy is particularly pronounced because teachers are expected to adapt to new curricular and pedagogical demands while continuing to meet performance expectations. Recent validation of the Teachers’ Sense of Efficacy Scale in Vietnam provides further support for using this construct in studies of teacher work and performance (Ho et al., 2023). Based on this literature, self-efficacy is treated in the present study as a key individual resource likely to positively predict job performance.

### 2.3 Resilience and job performance

Resilience refers to the capacity to adapt positively and sustain professional commitment under adverse or changing conditions. In teaching, resilience is not simply a fixed personal trait, but a dynamic process through which teachers cope with pressure, recover from setbacks, and continue functioning effectively in demanding environments (Beltman et al., 2011). Previous studies show that resilient teachers are better able to preserve motivation, regulate stress, and maintain professional engagement, especially during the early stages of their careers or in contexts of institutional uncertainty (Gu and Li, 2013; Mansfield et al., 2014). Reflective practice has also been identified as a key mechanism through which resilience develops over time (Leroux and Théorêt, 2014).

This construct is highly relevant in reforming educational environments, where teachers must navigate continuous adjustment and heightened expectations. In Vietnam, Trang and Thang (2023) developed and validated a four-factor model of teacher resilience, providing a contextually grounded basis for examining resilience in relation to teacher outcomes. In the present study, resilience is conceptualized as a protective psychological resource that may help sustain teachers’ job performance under reform-related and occupational pressures.

### 2.4 Principal support and work environment

Teacher performance is not shaped by personal resources alone. It is also influenced by the organizational and relational conditions created by school leadership. Principal support refers to teachers’ perceptions that school leaders provide guidance, understanding, communication, and practical support for their professional work. Studies have shown that supportive leadership is associated with stronger teacher commitment, improved work climate, and higher professional satisfaction (Boyd et al., 2011; Chong et al., 2015). Earlier work on school environments similarly highlighted the importance of workplace conditions and administrative climate for teachers’ perceptions of professional functioning (Huang, 2001; Kloep and Tarifa, 1994).

Research on transformational and distributed leadership further suggests that when school leaders empower teachers, communicate openly, and share responsibility, teachers are more likely to experience professional trust, autonomy, and psychological empowerment (García Torres, 2019; Leithwood and Sun, 2012; Liu and Werblow, 2019; Sun and Xia, 2018; Yao et al., 2020). Collegial support and collaborative work environments also function as important organizational resources. Studies across educational levels have shown that supportive climates, professional sharing, and social capital can strengthen teacher functioning and promote professional development (Abu Taleb, 2013; Clement and Vandenberghe, 2000; Edinger and Edinger, 2018; Hewett and La Paro, 2020; Kraft and Papay, 2014; Raza, 2010). For this reason, principal support is included in this study as a school-level factor expected to be positively associated with teachers’ job performance.

### 2.5 Burnout and job performance

Burnout is a psychological syndrome characterized by emotional exhaustion, depersonalization, and reduced personal accomplishment. In the teaching profession, burnout is widely recognized as one of the most important inhibiting factors undermining professional engagement and effectiveness. Prior research has linked burnout to lower motivation, reduced commitment, emotional withdrawal, and stronger intentions to leave the profession (Anastasiou and Belios, 2020; Capone and Petrillo, 2020; Gursel et al., 2002).

The literature also indicates that burnout among teachers is often associated not only with instructional challenges, but also with time pressure, administrative burden, lack of autonomy, and weak organizational support (Molero Jurado et al., 2019; Sarros and Sarros, 1992; Skaalvik and Skaalvik, 2014, 2017). These findings are particularly relevant in education systems undergoing reform, where documentation requirements, accountability demands, and implementation pressures may intensify teachers’ work strain. In this study, burnout is conceptualized as a negative occupational condition that is expected to be inversely related to job performance.

### 2.6 Broader implications for classroom and school functioning

Although the present study does not directly measure teacher-student relationships or collective efficacy, the broader literature suggests that the psychological and organizational conditions examined here may have implications beyond individual teacher performance. Teachers with stronger efficacy and more positive working conditions are often better positioned to build constructive classroom climates and sustain more positive interactions with students (Hur et al., 2016; Lavy and Bocker, 2018; Lee and Quek, 2018; Spilt et al., 2011; Yoon, 2002). Similarly, school climate, collective efficacy, and the use of performance information have been linked to teachers’ broader professional functioning and sense of effectiveness (Buonomo et al., 2020; Liu et al., 2018; Liu and Ramsey, 2008; Shaukat et al., 2019; Stephanou et al., 2013). These studies help situate the present model within a wider ecosystem of teacher work, even though the empirical focus of this article remains on self-efficacy, resilience, principal support, burnout, and job performance.

Taken together, the literature reviewed above suggests that teacher job performance is likely to be associated with both enabling and constraining factors. Self-efficacy and resilience represent internal professional resources that may strengthen teachers’ capacity to sustain effective work, while principal support reflects an external organizational condition that may facilitate professional functioning. By contrast, burnout represents a constraining factor that may weaken performance. On this basis, the present study examines how these four variables are related to the job performance of secondary school teachers in Vietnam and uses an explanatory sequential mixed-methods design to better understand not only the statistical patterns, but also how these relationships are experienced and interpreted in practice.

## 3 Methodology

### 3.1 Research design and methodological rationale

This study was grounded in pragmatism, a philosophical stance that supports the use of both quantitative and qualitative approaches when they are needed to address a research problem comprehensively. In line with this perspective, the study adopted a mixed-methods design to examine how individual and organizational factors were associated with the job performance of secondary school teachers in Vietnam. Mixed methods research is particularly appropriate when neither quantitative nor qualitative evidence alone is sufficient to capture both the general patterns and the contextual meanings of a phenomenon (Johnson and Onwuegbuzie, 2004; Johnson et al., 2007).

More specifically, the study employed an explanatory sequential design (QUAN → qual) (Ivankova et al., 2006). In the first phase, quantitative data were collected and analyzed to identify the statistical relationships among the focal variables. In the second phase, qualitative interviews were conducted to provide deeper insight into how teachers understood and experienced the patterns observed in the quantitative results. This design was chosen because the study aimed not only to identify predictive relationships, but also to understand how these relationships were interpreted in the context of educational reform and everyday school life. Thus, the qualitative phase served an explanatory and elaborative function rather than an independent exploratory one (Guest, 2013; Hanson et al., 2005).

### 3.2 Phase 1: sample and quantitative data collection procedures

The target population consisted of teachers working in lower and upper secondary schools in Vietnam. Because the study aimed to capture variation across institutional and contextual conditions, a stratified sampling strategy was employed. The sampling design was structured around three criteria: school sector, geographical area, and years of teaching experience. School sector was included to ensure that both governance contexts public and private schools were represented in the sample, while geographical area and teaching experience were included because they were theoretically relevant to differences in working conditions, professional adaptation, and school management environments.

At the school recruitment stage, the study focused on two educational settings, Ho Chi Minh City and the Mekong Delta, in order to capture variation between a major urban center and a regional context. Within these two settings, questionnaires were distributed to teachers from both public and private secondary schools through a combination of online and paper-based administration over a three-month period. To avoid overrepresentation of a single governance context, the sampling process was organized so that both school sectors were meaningfully represented in the achieved sample. Of the 536 valid responses retained for analysis, 311 teachers were from public schools (58.0%) and 225 were from private schools (42.0%). This distribution allowed the study to compare and interpret teachers’ responses across two distinct institutional environments without attempting to reproduce a nationally proportional sector distribution.

A total of 600 questionnaires were distributed, 565 were returned, and 536 valid responses were retained after data cleaning. Responses were excluded if they were substantially incomplete or showed no response variance across items.

Table 1 presents the composition of the achieved sample. Female teachers accounted for 70.2% of respondents, teachers with 5–15 years of experience formed the largest experience group (46.6%), and respondents were drawn from both Ho Chi Minh City (56.0%) and the Mekong Delta (44.0%). The predominance of female respondents is substantively plausible because teaching, especially in general education, has long been characterized as a female-majority profession in many educational settings. In this study, however, the gender distribution is reported primarily to describe the achieved sample, not to imply exact proportional correspondence with the national secondary-school workforce. In addition, the inclusion of teachers from public schools (58.0%) and private schools (42.0%) ensured that the study captured variation across two important governance settings in Vietnamese secondary education. Rather than claiming that this sample mirrors the exact national workforce structure, the present study treats it as an analytically diverse sample designed to reflect meaningful institutional and demographic variation relevant to the study objectives.

**Table 1:** Demographic characteristics of the quantitative sample (*N =* 536).

Characteristics	Category	Frequency (*n*)	Percentage (%)
Gender	Male	160	29.8
	Female	376	70.2
Experience	Under 5 years	106	19.8
	5–15 years	250	46.6
	Over 15 years	180	33.6
Qualification	Bachelor’s degree	428	79.9
	Master’s/Doctoral degree	108	20.1
Region	Ho Chi Minh City	300	56
	Mekong Delta	236	44
School sector	Public	311	58
	Private	225	42

### 3.3 Measurement instruments and quantitative scale assessment

The structured questionnaire measured five focal constructs in the analytical model: self-efficacy, principal support, resilience, burnout, and job performance. The instrument was developed through adaptation of previously validated scales, including the Vietnamese validation of the Teachers’ Sense of Efficacy Scale (Ho et al., 2023) and the Vietnamese teacher resilience scale (Trang and Thang, 2023). To strengthen semantic equivalence, a back-translation procedure was carried out by two independent bilingual experts. All items were rated on a five-point Likert scale ranging from 1 = strongly disagree to 5 = strongly agree.

Before the regression analysis was conducted, the internal consistency and convergent validity of the scales were assessed. Cronbach’s alpha was used to evaluate reliability, while composite reliability and average variance extracted were used as additional indicators of construct quality (Table 2).

**Table 2:** Reliability and convergent validity of the scales (*N =* 536).

Construct	Number of items	Cronbach’s Alpha	CR	AVE
Self-Efficacy (SE)	6	0.912	0.920	0.655
Principal Support (PS)	5	0.865	0.872	0.594
Resilience (RES)	4	0.878	0.885	0.638
Burnout (BO)	5	0.905	0.910	0.682
Job Performance (JP)	6	0.896	0.902	0.608

The results indicate that all scales achieved satisfactory reliability and convergent validity. Cronbach’s alpha coefficients ranged from 0.865 to 0.912, well above the conventional threshold of 0.70. Composite reliability values ranged from 0.872 to 0.920, and all average variance extracted values exceeded 0.50, indicating that the observed items captured a substantial proportion of variance in their intended constructs. These results provided an acceptable basis for computing composite scores and proceeding with the regression analysis.

### 3.4 Phase 2: qualitative data collection procedures

The qualitative phase was designed to explain and elaborate the quantitative findings. Interview participants were selected from among the survey respondents using purposive sampling combined with maximum variation logic. Selection was based primarily on variation in job performance scores, together with variation in teaching experience, gender, geographical area, and school sector where possible. This strategy enabled the study to include participants representing different professional circumstances and performance profiles, thereby increasing the explanatory value of the qualitative dataset.

To operationalize this process, the 536 survey respondents were grouped according to their job performance scores into three broad categories: low-to-moderate, moderate-to-high, and high. From these groups, 16 teachers were invited to participate in semi-structured interviews (Table 3).

**Table 3:** Characteristics of qualitative interview participants (*N =* 16).

Code	Gender	Teaching experience	Working region	Performance group
T01–T04	2 Male, 2 Female	Under 5 years	Mekong Delta (2), HCMC (2)	Low-Moderate
T05–T10	1 Male, 5 Female	5–15 years	Mekong Delta (3), HCMC (3)	Moderate-High
T11–T16	4 Male, 2 Female	Over 15 years	Mekong Delta (2), HCMC (4)	High

The qualitative sample was intended to provide a broad range of perspectives rather than numerical representativeness. The inclusion of early-career teachers made it possible to explore professional strain and adaptation, while the inclusion of highly experienced teachers provided insight into how sustained performance and resilience were maintained over time.

The semi-structured interview guide was developed from the quantitative findings. In particular, the interview questions were designed to clarify why self-efficacy emerged as the strongest predictor, how principal support operated in everyday school practice, and why burnout remained negatively associated with teachers’ job performance. Interviews lasted approximately 45 to 60 min and were conducted either face-to-face or online, depending on participants’ availability and location. All interviews were audio-recorded with participants’ written consent and subsequently transcribed verbatim for analysis.

### 3.5 Data analysis and integration procedures

Quantitative data were analyzed using descriptive statistics, Pearson correlation analysis, and multiple linear regression. Descriptive statistics were first calculated to examine central tendency, dispersion, and distributional characteristics of the data. Pearson correlations were then used to assess the direction and strength of associations among the study variables. Finally, multiple regression analysis was conducted to estimate the predictive relationships between self-efficacy, principal support, resilience, burnout, and job performance. To strengthen the robustness of the quantitative strand, multicollinearity was examined using the variance inflation factor, and common method bias was preliminarily assessed using Harman’s single-factor test.

Qualitative data were analyzed using thematic analysis with support from NVivo software. The coding and theme-development process involved two researchers from the author team, who worked collaboratively throughout the analytic process. In the first stage, all interview transcripts were read repeatedly to achieve familiarity with the dataset and to identify meaning units relevant to teachers’ experiences of performance, support, pressure, and coping. In the second stage, the two researchers independently conducted initial coding on a subset of transcripts and then compared their coding decisions in order to refine code definitions, clarify category boundaries, and improve coding consistency. Differences in interpretation were discussed until agreement was reached, after which the refined coding framework was applied to the full dataset.

Theme development followed a hybrid inductive-deductive logic. The initial codes were generated directly from participants’ accounts, allowing themes to emerge from the interview data rather than being imposed *a priori*. At the same time, the interpretation and refinement of these themes were informed by the study’s conceptual framework, particularly the focal constructs of self-efficacy, principal support, resilience, burnout, and job performance. After the initial coding stage, related codes were grouped into broader categories, which were then reviewed, collapsed, or differentiated into candidate themes. These themes were repeatedly checked against the full dataset to ensure internal coherence, conceptual distinction, and adequate grounding in participants’ narratives. Representative quotations were then selected to illustrate the final thematic structure in the Results section.

Integration followed the principles outlined by Fetters et al. (2013) and occurred at three points. First, the study used a connecting strategy because interview participants were selected from the pool of survey respondents. Second, it used a building strategy because the quantitative findings informed the content of the qualitative interview guide. Third, it used a merging strategy at the interpretation stage, where quantitative results and qualitative themes were brought together through a joint display. The purpose of this integration was to clarify and elaborate the statistical relationships identified in the regression model and to show how these relationships were experienced and interpreted by teachers in practice.

### 3.6 Quality criteria, trustworthiness, and research ethics

For the quantitative strand, methodological quality was addressed through established indicators of reliability and construct quality, including Cronbach’s alpha, composite reliability, and average variance extracted. In addition, diagnostic checks for regression analysis, including variance inflation factor and Harman’s single-factor test, were conducted to strengthen the robustness of the statistical results.

For the qualitative strand, trustworthiness was addressed in terms of credibility, dependability, confirmability, and transferability (Harrison et al., 2020; O’Cathain et al., 2008; Tariq and Woodman, 2013). Credibility was enhanced through careful verbatim transcription, repeated engagement with the interview data, and member checking during the interview and interpretation process. Dependability was supported by maintaining a transparent analytic procedure, including iterative refinement of the codebook, documentation of coding decisions, and ongoing discussion between the two researchers involved in the coding process. Confirmability was strengthened by grounding interpretations in verbatim quotations and by comparing emerging themes against the raw data throughout the analysis. Transferability was addressed by providing sufficient contextual detail about participants, school settings, and the Vietnamese reform environment so that readers can assess the relevance of the findings to comparable educational contexts.

The study adhered to standard ethical principles for research involving human participants. Participation was voluntary, informed consent was obtained prior to data collection, and respondents were assured that their identities would remain confidential. Survey data were anonymized, and interview participants were assigned codes rather than named identifiers. All data were used solely for academic purposes and handled in ways that protected participants’ privacy and professional standing.

## 4 Results

### 4.1 Descriptive statistics and data distribution

Before testing the study hypotheses through correlation and regression analyses, descriptive statistics were examined to assess central tendency, dispersion, and the distributional characteristics of the data collected from 536 teachers. The results are presented in Table 4.

**Table 4:** Descriptive statistics of the main constructs (*N =* 536).

Constructs	Mean	Standard deviation (SD)	Skewness	Kurtosis
Self-Efficacy (SE)	3.92	0.74	−0.48	0.28
Principal Support (PS)	3.45	0.89	−0.35	0.11
Resilience (RES)	3.75	0.78	−0.45	0.18
Burnout (BO)	2.82	1.02	0.38	−0.25
Job Performance (JP)	3.98	0.70	−0.58	0.42

As shown in Table 4, job performance recorded the highest mean score (M = 3.98, SD = 0.70), followed by self-efficacy (M = 3.92, SD = 0.74). These values suggest that respondents generally perceived themselves as functioning effectively in their professional roles while also expressing relatively strong confidence in their instructional capabilities. Resilience also remained at a favorable level (M = 3.75, SD = 0.78), indicating that many teachers viewed themselves as able to cope with professional pressures and adapt to work-related challenges.

By contrast, principal support recorded a more moderate mean score (M = 3.45, SD = 0.89), suggesting that organizational support from school leaders was present but less strongly perceived than teachers’ own internal professional resources. Burnout had the lowest mean score (M = 2.82, SD = 1.02), but also the highest standard deviation, indicating greater variation across respondents. This pattern suggests that burnout was not experienced uniformly and therefore warranted deeper qualitative exploration. With respect to distributional assumptions, skewness values ranged from −0.58 to 0.38 and kurtosis values ranged from −0.25 to 0.42. All values fell well within acceptable thresholds, suggesting that the data did not show serious departures from normality and were suitable for subsequent parametric analyses.

### 4.2 Pearson correlation analysis

Following the descriptive analysis, Pearson correlation coefficients were calculated to examine the strength and direction of associations among the study variables. The results are presented in Table 5.

**Table 5:** Pearson correlation matrix (*N =* 536).

Variables	1	2	3	4	5
(1) Self-Efficacy (SE)	1				
(2) Principal Support (PS)	0.38**	1			
(3) Resilience (RES)	0.62**	0.32**	1		
(4) Burnout (BO)	−0.47**	−0.52**	−0.58**	1	
(5) Job Performance (JP)	0.67**	0.42**	0.54**	−0.50**	1

**p < 0.01 (two-tailed test).

The correlation matrix indicates that all focal variables were significantly associated at the *p <* 0.01 level. Job performance showed the strongest positive association with self-efficacy (r = 0.67), followed by resilience (r = 0.54) and principal support (r = 0.42). These findings suggest that both internal professional resources and organizational support are meaningfully related to teachers’ perceived work performance.

Burnout, in contrast, was negatively associated with all positive variables, including self-efficacy (r = −0.47), principal support (r = −0.52), resilience (r = −0.58), and job performance (r = −0.50). This pattern indicates that greater burnout tended to coincide with weaker perceived professional functioning and lower psychological resources.

Importantly, the correlations among the independent variables remained below the commonly used threshold of 0.70, suggesting that serious multicollinearity was unlikely and that it was appropriate to proceed with multiple regression analysis.

### 4.3 Multiple linear regression analysis

To examine the predictive relationships between the focal variables and teachers’ job performance, a multiple linear regression model was estimated with job performance as the dependent variable. The results are shown in Table 6.

**Table 6:** Linear regression results (dependent variable: job performance).

Independent variables	Standardized *β* coefficient	*t*-value	*p*-value	VIF
Constant		5.14	< 0.001	
Self-Efficacy (SE)	0.38	8.25	< 0.001	1.88
Resilience (RES)	0.21	5.32	< 0.001	1.68
Principal Support (PS)	0.16	4.45	< 0.001	1.45
Burnout (BO)	−0.14	−3.68	< 0.001	1.92

Adjusted R^2^ = 0.562; F = 172.4, *p <* 0.001; Durbin-Watson = 1.95.

The regression model was statistically significant and explained 56.2% of the variance in job performance. The Durbin-Watson statistic suggested no serious autocorrelation, while all VIF values were below 2.0, indicating that multicollinearity was not a major concern.

Among the predictors, self-efficacy emerged as the strongest positive predictor of job performance. Resilience was the second strongest predictor, followed by principal support. Burnout was negatively associated with job performance. Overall, the results suggest that teachers’ job performance was more strongly predicted by internal professional resources, particularly self-efficacy, while organizational support and burnout also contributed meaningfully to the model.

### 4.4 Qualitative findings: themes explaining the statistical patterns

The qualitative phase was conducted to provide contextual explanations for the statistical relationships identified in the quantitative strand. Thematic analysis of 16 semi-structured interviews generated four main themes that reflected both enabling and constraining conditions affecting teachers’ job performance (Table 7).

**Table 7:** Thematic analysis of interview data (*N =* 16).

Main themes	Sub-themes	Verbatim quotes	Frequency
Core Competence	Pedagogical autonomy and professional mastery	“When I master technology and new teaching methods, managing my classroom becomes much easier.” (T14)	15/16
Supportive Leadership	Listening, guidance, and delegation	“Clear understanding and delegation from department heads and school leaders are my greatest motivation.” (T08)	12/16
Systemic Barriers	Administrative burden	“The volume of paperwork and performance documentation is draining the enthusiasm of young teachers like us.” (T02)	14/16
Psychological Buffer	Peer support and collegial sharing	“Professional sharing within the department helps me recover after unsuccessful lessons.” (T06)	11/16

The most prominent theme was core competence. Teachers frequently described professional confidence as rooted in pedagogical mastery, adaptability, and the ability to use teaching methods and technology effectively. This theme provides contextual support for the quantitative finding that self-efficacy was the strongest predictor of job performance.

The theme of supportive leadership highlighted the importance of school leaders who listened, communicated clearly, and delegated responsibility in ways that enabled teachers to focus on professional tasks. Rather than emphasizing formal authority, participants associated leadership support with understanding, trust, and practical guidance. This finding helps explain why principal support remained a significant positive predictor in the regression model.

The theme of systemic barriers centered on the burden of paperwork, reporting requirements, and administrative procedures. Participants commonly described these demands as energy-depleting and as competing with instructional responsibilities. This pattern helps clarify the negative statistical association between burnout and job performance.

Finally, psychological buffer captured the role of collegial support, especially informal peer discussion and professional sharing. Teachers described these networks as important sources of emotional recovery and practical coping, particularly during difficult teaching periods. This theme provides qualitative support for the positive role of resilience and the social conditions that may help sustain it.

### 4.5 Integration of quantitative and qualitative findings

In the explanatory sequential design, integration was used to clarify how the quantitative relationships were experienced and interpreted in practice. A joint display was constructed to align the regression findings with the qualitative themes (Table 8).

**Table 8:** Integration of quantitative and qualitative findings (joint display).

Variables/factors	Quantitative results (*N =* 536)	Qualitative explanation (*N =* 16)	Meta-inference
Self-Efficacy (SE)	Strongest predictor (*β* = 0.38, *p <* 0.001)	Teachers linked strong performance to pedagogical competence, adaptability, and mastery of teaching methods and technology.	Self-efficacy represents a core professional resource supporting teachers’ work performance.
Resilience (RES)	Second strongest predictor (*β* = 0.21, *p <* 0.001)	Teachers described coping capacity as strengthened through reflective adaptation and collegial support.	Resilience functions as a protective resource that helps teachers sustain performance under pressure.
Principal Support (PS)	Positive predictor (*β* = 0.16, *p <* 0.001)	Teachers emphasized the value of leaders who listened, guided, and delegated appropriately.	Principal support contributes to performance by shaping a more supportive working environment.
Burnout (BO)	Negative predictor (*β* = −0.14, *p <* 0.001)	Teachers associated exhaustion mainly with administrative workload and documentation demands.	Burnout operates as a constraining condition that weakens teachers’ professional functioning.

The integrated findings show substantial convergence between the two strands of the study. Quantitatively, self-efficacy emerged as the strongest predictor of job performance; qualitatively, teachers described professional mastery and pedagogical confidence as central to their sense of effectiveness. Similarly, resilience was positively associated with performance in the regression model, while interview data showed that teachers relied on adaptation, reflection, and collegial support to sustain themselves through demanding work conditions.

Principal support also remained significant across both strands. While the quantitative analysis showed a positive predictive relationship, the qualitative findings clarified that this support was experienced less as formal managerial control and more as practical guidance, trust, and open communication. Burnout, in turn, was negatively associated with job performance, and the interviews clarified that this exhaustion was linked primarily to administrative and non-instructional pressures rather than to teaching itself.

Taken together, the mixed-methods findings suggest that teachers’ job performance in the Vietnamese context is shaped by the interplay between internal professional resources and organizational conditions. Performance appears to be strengthened when teachers possess strong self-efficacy and resilience and when school leadership provides meaningful support, but weakened when administrative pressure intensifies burnout.

## 5 Discussion

The purpose of this study was to examine how self-efficacy, principal support, resilience, and burnout were associated with the job performance of secondary school teachers in Vietnam, and to use qualitative evidence to better understand how these statistical patterns were experienced in practice. Overall, the findings suggest that teachers’ job performance is related to both internal psychological resources and organizational working conditions, with self-efficacy emerging as the strongest predictor in the model.

One notable finding was the positive association between self-efficacy and job performance. This result is consistent with previous research indicating that teachers who feel more capable in instructional and classroom-management tasks tend to function more effectively in demanding professional environments (Burić and Kim, 2021; Canrinus et al., 2012; Klassen and Chiu, 2010). The qualitative findings add further interpretive depth by suggesting that teachers in this study did not understand confidence as an abstract belief alone, but as something grounded in pedagogical competence, adaptability, and the ability to manage classroom demands in practice. In the Vietnamese reform context, where teachers are expected to adjust to new curricular expectations and instructional requirements, this pattern may indicate that self-efficacy is especially relevant to sustaining everyday professional functioning.

Resilience was also positively associated with job performance. This finding is in line with the view that resilience is better understood as a dynamic professional resource than as a fixed personal trait (Beltman et al., 2011; Gu and Li, 2013; Mansfield et al., 2014). The interview data suggest that teachers’ coping capacity may be supported not only by individual mindset, but also by collegial interaction and opportunities for professional sharing. This interpretation is consistent with more recent work emphasizing the importance of collaborative culture and teacher interaction in shaping work-related outcomes. For example, Meredith et al. (2023) highlight the importance of collaborative culture for teachers’ job satisfaction and affective commitment, while Liu et al. (2024) point to the moderating role of teacher collaboration in the association between job satisfaction and job performance. Although collaboration was not directly measured in the present study, the qualitative evidence suggests that peer support may be one of the contextual conditions through which resilience is sustained in practice.

At the organizational level, principal support was positively associated with job performance. This finding broadly aligns with prior studies showing that supportive leadership contributes to teachers’ work environment and professional engagement (Boyd et al., 2011; Chong et al., 2015; Yao et al., 2020). The qualitative strand helps refine this interpretation by showing that teachers appeared to value leadership less as formal authority and more as a form of relational and practical support expressed through listening, trust, clarity, and appropriate delegation. In this respect, the present findings are also compatible with more recent scholarship emphasizing the role of collective and collaborative school culture. Battal and Demirkasimoğlu (2025), for example, argue for the importance of collective teacher culture in linking autonomy, job satisfaction, and job performance. While the present study did not directly measure autonomy or collective culture as separate variables, the interview narratives suggest that supportive leadership may matter partly because it creates more workable and professionally enabling conditions for teachers.

Burnout was the only variable negatively associated with job performance. This result is consistent with the broader literature linking burnout to emotional exhaustion, reduced engagement, and weaker professional functioning (Anastasiou and Belios, 2020; Capone and Petrillo, 2020; Molero Jurado et al., 2019). However, the qualitative findings should be interpreted carefully. They do not establish the definitive causes of burnout; rather, they suggest that many teachers in this sample associated exhaustion more strongly with paperwork, documentation, and administrative requirements than with teaching itself. This interpretation resonates with the work of Skaalvik and Skaalvik (2016, 2017), who consistently identify non-instructional pressure and limited autonomy as important sources of teacher strain. In the Vietnamese reform context, this pattern may be particularly salient because policy change can expand both professional expectations and administrative obligations at the same time.

Taken together, the findings suggest that teacher job performance in a reform-oriented education system may be better understood as the outcome of an interaction between professional capacity and organizational conditions rather than as the result of individual characteristics alone. Self-efficacy and resilience appear to function as important internal resources, whereas principal support and administrative burden reflect organizational features that may either support or constrain teachers’ work. From this perspective, the Vietnamese case offers a useful empirical context for understanding teacher work in other centralized or rapidly changing educational systems, especially where reform implementation places simultaneous demands on instructional adaptation and administrative compliance.

At the same time, the findings should be interpreted with appropriate caution. Because the quantitative strand was cross-sectional, the study does not support firm causal claims. Its contribution lies more modestly in identifying statistically meaningful relationships and using qualitative evidence to provide context-sensitive interpretations of how these relationships may operate in practice. Viewed in this way, the mixed-methods design strengthens the explanatory richness of the study without implying definitive causal inference.

## 6 Conclusion and implications

### 6.1 Conclusion

This study employed an explanatory sequential mixed-methods design to examine how self-efficacy, principal support, resilience, and burnout were associated with the job performance of secondary school teachers in Vietnam. By combining survey data from 536 teachers with in-depth interviews with 16 participants, the study provided both statistical evidence and contextual insight into how these relationships may operate in practice.

Overall, the findings suggest that self-efficacy was the strongest positive predictor of job performance, followed by resilience and principal support, whereas burnout was negatively associated with performance. The qualitative findings add interpretive depth to these statistical patterns. In particular, teachers tended to associate stronger performance with pedagogical competence, adaptability, and confidence in managing instructional demands. They also described supportive leadership and collegial sharing as important conditions that helped sustain their professional functioning. By contrast, many participants linked feelings of exhaustion more closely to administrative workload and documentation pressure than to teaching itself.

Taken together, the findings suggest that teacher job performance in the Vietnamese context is related not only to individual psychological resources, but also to the organizational conditions under which teachers work. In this sense, the study supports the view that teacher performance is embedded in a broader professional and institutional environment, especially in periods of educational reform.

### 6.2 Theoretical and practical implications

#### 6.2.1 Theoretical implications

This study contributes to the literature by supporting an integrated view of teacher job performance as a multidimensional outcome associated with both psychological and organizational factors. More specifically, the findings suggest that self-efficacy and resilience may function as important internal professional resources, while principal support and burnout reflect organizational conditions that can either support or constrain teachers’ work. The Vietnamese case also adds contextual value to international discussions of teacher work by showing how these relationships may unfold in a reform-oriented education system characterized by strong policy pressure and expanding professional demands.

In addition, the mixed-methods design contributes by moving beyond statistical pattern identification alone. Although the study does not establish causality, the qualitative strand helps clarify how teachers themselves interpret the conditions associated with stronger or weaker job performance. This interpretive layer strengthens the explanatory value of the findings and may be especially useful for understanding teacher work in similar centralized or rapidly changing educational settings.

#### 6.2.2 Practical implications

The findings also suggest several practical implications for school leaders and policymakers.

First, efforts to improve teacher performance may benefit from paying greater attention to teachers’ sense of professional competence. Because self-efficacy showed the strongest positive association with job performance, professional development initiatives should not focus only on compliance or evaluation, but also on strengthening teachers’ instructional confidence, pedagogical adaptability, and capacity to respond to changing classroom demands.

Second, leadership practices appear to matter not simply as formal managerial functions, but as forms of relational and practical support. The qualitative findings suggest that teachers particularly value leaders who listen, communicate clearly, and provide appropriate guidance and delegation. This implies that leadership development in schools may be more effective when it emphasizes supportive communication, trust, and professional enablement rather than administrative control alone.

Third, the findings suggest that administrative workload deserves closer policy attention. While this study cannot claim that documentation demands are the sole or definitive cause of burnout, the interview data indicate that many teachers experience administrative pressure as a significant source of strain. Simplifying reporting procedures, streamlining documentation requirements, and reducing unnecessary non-instructional tasks may therefore help create more supportive working conditions for teachers.

Finally, schools may benefit from strengthening collegial support structures. Peer sharing, collaborative discussion, and internal professional support appeared in the qualitative data as helpful resources for coping with pressure and sustaining resilience. Building and maintaining such collaborative environments may therefore contribute indirectly to stronger professional functioning.

### 6.3 Limitations and future research directions

This study has several limitations. First, the quantitative strand relied on cross-sectional data, which limits the ability to make temporal or causal claims. Second, the study used self-reported measures, which may be subject to response bias. Third, although the qualitative phase added interpretive depth, the findings remain shaped by the institutional and regional contexts included in the study.

Future research could build on these findings in several ways. Longitudinal studies would be useful for examining how self-efficacy, resilience, burnout, and job performance develop over time, especially during periods of educational change. Comparative studies across public and private school sectors, across regions, or across educational levels could also provide a more differentiated understanding of how institutional context shapes these relationships. In addition, future mixed-methods research could examine more directly the role of collaborative culture, administrative reform, and school-level leadership practices in shaping teachers’ professional experience and performance.

## Data Availability

The original contributions presented in the study are included in the article/supplementary material, further inquiries can be directed to the corresponding author.
